# Five new species of *Mydaea* from China (Diptera, Muscidae)

**DOI:** 10.3897/zookeys.897.39232

**Published:** 2019-12-09

**Authors:** Jing Du, Bo Hao, Wanqi Xue, Chuntian Zhang

**Affiliations:** 1 College of Life Science, Shenyang Normal University, Shenyang 110034, China Shenyang Normal University Shenyang China; 2 Institute of Entomology, Shenyang Normal University, Shenyang 110034, China Shenyang Normal University Shenyang China

**Keywords:** Calyptratae, description, key, Muscoidea, taxonomy

## Abstract

Five new species of *Mydaea* are described from China, namely *M. adhesipeda* Xue, **sp. nov.**, *M. combiniseriata* Xue, **sp. nov.**, *M. qingyuanensis* Xue, **sp. nov.**, *M. quinquiseta* Xue, **sp. nov.**, *M. wusuensis* Xue, **sp. nov.**, and an addendum to the key of the *Mydaea* in China is given.

## Introduction

*Mydaea* Robineau-Desvoidy, 1830 is a genus in the subfamily Mydaeinae (Diptera, Muscidae). It comprises approximately 120 species worldwide. About 100 species were recorded from the Palaearctic, 35 from the Nearctic, 26 from the Neotropical, nine from the Oriental, and two species from the Afrotropical regions (Hennig 1957; Huckett 1965; van Emden 1965; Vockeroth 1972; Pont 1977, 1980, 1986, 1989; Shinonaga 2003; Carvalho et al. 2005). Thirty species were reported from China, approximately one-fifth of the species worldwide (Ma et al. 1986; Xue 1992; Xue and Chao 1996; Feng 2000a, b, 2003; Xue and Tian 2012, 2014). During the study of our collection in recent years, five new species were found in the mountains from Liaoning, Heilongjiang, and Sichuan provinces and from the Ningxia Hui Autonomous Region of China. Descriptions, figures, and an addendum to the key to the Chinese species of the genus *Mydaea* are provided.

## Methods and materials

The specimens examined for this study were collected by sweeping. The genitalia were detached from the abdomen, bleached by heating in a 10% NaOH solution (approximately 100 °C) for about 20 min, placed in a droplet of glycerol, and examined using an Olympus SZX7 stereomicroscope. After examination, the genitalia were stored in a small plastic vial filled with glycerin and pinned with the specimen. The type material is deposited in the Insect Collection, Shenyang Normal University, Shenyang, China (**SYNU**).

The morphological terminology follows that of Cumming and Wood (2017). Absolute measurements of the body length are in millimeters (mm). Adhesive hairs are special hair-like setae modified for climbing or gripping. The following abbreviations are used for various morphological structures: *acr*, acrostichal setae; *prst-acr*, presutural acrostichal setae; *dc*, dorsocentral setae; *ial*, intra-alar setae; *pra*, prealar setae; *av*, anteroventral setae; *ad*, anterodorsal setae; *pd*, posterodorsal setae; *p*, posterior setae; *pv*, posteroventral setae.

## Results

### Mydaea

Taxon classificationAnimaliaDipteraMuscidae

Robineau-Desvoidy, 1830

3A7D6B5C-12E3-5155-9B79-4D5EFF50E821

Mydaea Robineau-Desvoidy, 1830: 479. Type species: Mydaea scutellaris Robineau-Desvoidy, 1830 (by subsequent designation of Coquillett 1901).
Xenomydaea Malloch, 1920: 144. Type species: Xenomydaea buccata Malloch, 1920 (by original designation).

#### Generic diagnosis.

**Figure 1.**
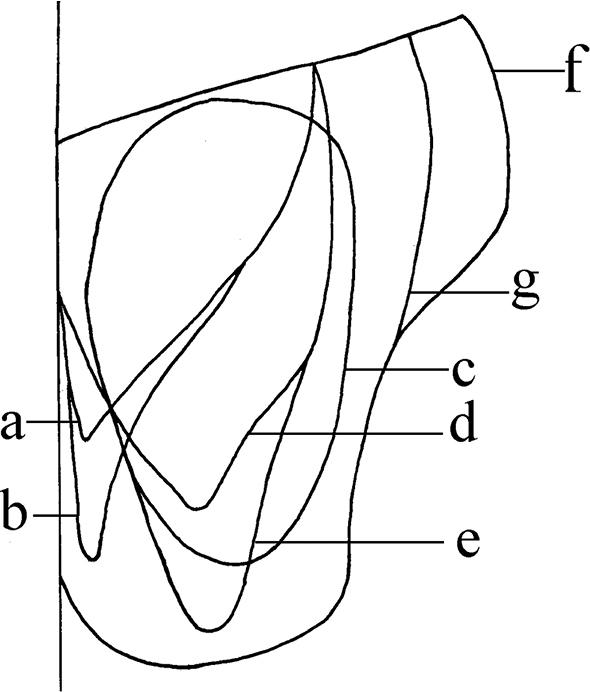
Cerci types of Chinese *Mydaea***a***M. subelecta* Feng, 2000; *M. gracilior* Xue, 1992 **b***M. jiuzhaigouensis* Feng & Deng, 2001; *M. nubila* Stein, 1916; *M. scolocerca* Feng, 2000 **c***Mydaea ancilloides* Xue, 1992; *M. jubiventera* Feng & Deng, 2001; *M. fuchaoi* Xue & Tian, 2012; *M. flavifemora* Feng, 2000; *M. kangdinga* Xue & Feng, 1992; *M. setifemur* Ringdahl, 1924 **d***M. brunneipennis* Wei, 1994; *Mydaea glaucina* Wei, 1994; *M. nigra* Wei, 1994; *M. shuensis* Feng, 2003; *M. minor* Ma & Wu, 1986; *M. sinensis* Ma & Cui, 1986; *M. franzosternita* Xue & Tian, 2014; *M. laxidetrita* Xue & Wang, 1992 **e***M. nigribasicosta* Xue & Feng, 1996; *M. latielecta* Xue, 1992; *M. tinctoscutaris* Xue, 1992 **f***M. affinis* Meade, 1891; *M. bideserta* Xue & Wang, 1992; *M. urbana* (Meigen, 1826) **g***M. brevis* Wei, 1994; *M. emeishanna* Feng & Deng, 2001; *M. discocerca* Feng, 2000; *M. minutiglaucina* Xue & Tian, 2012.

Meron and anepimeron bare; katepisternal setae 1+2; lower margin of posterior spiracle without row of setae; Sc bent bow-shaped; dorsal and ventral surfaces of radial node with hairs, M_1_ straight; lower calypter tongue-shaped; mid tibia with at least 2 *p*; hind coxa bare on posterior surface, hind tibia without *pd.* The cerci of all Chinese species have been divided into seven different kinds. We figure together these seven kinds to show there differences (Fig. 1).

##### Addendum to the key by Xue and Tian (2014) of *Mydaea* (males only)

**Table:** 

7	Scutellum yellow or with yellow basal part	**8**
–	Scutellum entirely black	**7a**
7a	Hind femur black	***M. wusuensis* Xue, sp. nov.**
–	Hind femur yellow or fuscous	**11**
8	Hind femur with *pv* in rows	**9**
–	Hind femur with *pv* on the base at most	**8a**
8a	Postpronotal lobe yellow	***M. tinctoscutaris* Xue, 1992**
–	Postpronotal lobe black	***M. quinquiseta* Xue, sp. nov.**
9	Anterior spiracle yellow	**9a**
–	Anterior spiracle fuscous	**10**
9a	2 *prst-acr*	***M. gracilior* Xue, 1992**
–	*prst-acr* absent	***M. qingyuanensis* Xue, sp. nov.**
10	Coxae, trochanters of fore leg and all tarsi fuscous	**10a**
–	All legs yellow	***M. kangdinga* Xue & Feng, 1992**
10a	Ventral surface of fore femur fuscous on basal half	***M. setifemur* Ringdahl, 1924**
–	Ventral surface of fore femur yellow on basal half	***M. combiniseriata* Xue, sp. nov.**
13	Frons subequal with anterior ocellus in width abdomen with shifting patches	***M. discocerca* Feng, 2000**
–	Frons about 2 times as wide as anterior ocellus; abdomen without shifting patch	**15**
14	*Pra* about 1/2 of posterior notopleural seta in length; wing brown, basal half of hind femur with *pv* obviously	**19**
–	*Pra* longer than posterior notopleural seta; wing yellow, hind femur with sparse and short *pv*	**20**
15	Antennal arista ciliated, the longest hair subequal with antennal postpedicel in width	**16**
–	Antennal arista short ciliated, the longest hair longer than antennal postpedicel in width	**17**
16	Parafacial about 1/2 of postpedicel in width	***M. brevis* Wei, 1994**
–	Parafacial subequal with postpedicel in width	**16a**
16a	Basicosta dark-brown, *pra* approximately 1.3 times as long as posterior notopleural seta; fore tibia without median *p*, ventral surface of fore tarsus without adhesive hairs	***M. fuchaoi* Xue & Tian, 2012**
–	Basicosta yellow, *pra* shorter than posterior notopleural seta; fore tibia with 1 median *p* , ventral surface of fore tarsus with adhesive hairs	***M. adhesipeda* Xue, sp. nov.**

### Mydaea adhesipeda

Taxon classificationAnimaliaDipteraMuscidae

Xue sp. nov.

595AD278-4A62-5D19-AE56-7B69202E9519

http://zoobank.org/C84CC490-8266-4ACD-96AC-2FE70E5E5058

#### Type material.

***Holotype*.** China, 1 ♂, Ningxia Hui Autonomous Region, Jingyuan, Liupan Mountains, 35°39'N, 106°34'E, alt. 2200 m, 3 July 2009, Zhiyuan Yao, (SYNU). ***Paratypes*.** 2♂♂, 5 ♀♀, same data as holotype.

#### Diagnosis.

*Post-dc* 4, scutellum dark black; basal and apical scutellar seta approximately as long as hind tibia; coxae and trochanters brown, tarsi dark brown, ventral surface of fore tarsus with adhesive hairs; femora and tibiae yellow; hind femur with *av* rows on distal half.

#### Description.

**Male.** Body length 8.0–8.5 mm. ***Head***: eyes bare, facets on upper half not enlarged. Frontal vitta black; lunule brown; genal and postgenal hairs entirely black; antenna fuscous, palpus black. Frons as wide as postpedicel, and slightly wider than distance between outer margins of posterior ocelli; fronto-orbital plate narrow, 2/5 of width of frons at middle; fronto-orbital plates, parafacials and genae covered with sparse gray pruinosity, parafacials as wide as or narrower than the width of postpedicel; genal height approximately 1/9 of eye height; lower face not projecting, vibrissal angle situated behind frontal angle in lateral view. Frontal setae with 7 or 8 pairs, upper 2 pairs short and situated on lower 4/5 of frons; proclinate orbital setae absent; ocellar setae approximately 2/3 of lower pair of frontal setae in length; postpedicel 3.5 times as long as wide; arista ciliated and longest hair approximately as long as width of postpedicel; palpi as long as prementum; prementum 2.5 times as long as wide, and covered with gray pruinosity; labellum extending to posterior part, and approximately 4/5 as long as palpi. ***Thorax***: ground color black, covered with sparse gray pruinosity and slightly shining; scutum with 4 black vittae, and the inner vitta extending to scutoscutellar suture; *acr* 0+1; *dc* 2+4; *ial* 0+2; *pra* slightly shorter than posterior notopleural seta; notopleuron with small hairs; scutellum black, basal and apical scutellar seta strong, approximately as long as hind tibia; basisternum of prosternum, anepimeron, meron, and katepimeron bare; katepisternal setae 1+2; 1 anepisternal seta; anterior and posterior spiracles yellow. ***Wings***: semi-hyaline; tegula dark brown; basicosta yellow; ventral surface of vein C with hairs; vein Sc bow-shaped; dorsal and ventral surfaces of radial node with hairs; the middle part of crossvein dm-m bent towards base of wing, areas around crossveins r-m and dm-m not clouded; R_4+5_ and M_1_ straight and diverging slightly distally; calypter yellowish, lower calypter tongue-shaped; halter knob yellow. ***Legs***: coxae and trochanters brown, tarsi dark brown, femora and tibiae yellow; ventral surface of fore tarsus with adhesive hairs, fore tibia with 1 median *p*; mid femur without *av* and 6 *pv* on basal half, 2 preapical *ad*, 3 *pd*, mid tibia with 2 or 3 (a few with 4) *pv*; hind femur with *av* rows on distal half, *av* short on basal half, without *pv*, hind tibia with 4 *av*, 2 *ad*, without apical *pv*; tarsi longer than tibiae; fore claws and pulvilli approximately 1.2 times as long as tarsomere 5, mid claws and pulvilli approximately as long as tarsomere 5, hind claws and pulvilli shorter than tarsomere 5. ***Abdomen***: Black in ground color, ovate in dorsal view, covered with gray pruinosity, both sides without color shifting patch, tergites 3 and 4 with a black median vitta, tergites 4 and 5 each with a complete row of posterior marginal setae, both sides of tergite 5 with 3 or 4 discal setae on anterior half; sternite 1 bare; middle part of lateral margin of cerci invaginated slightly in posterior view.

**Figure 2.**
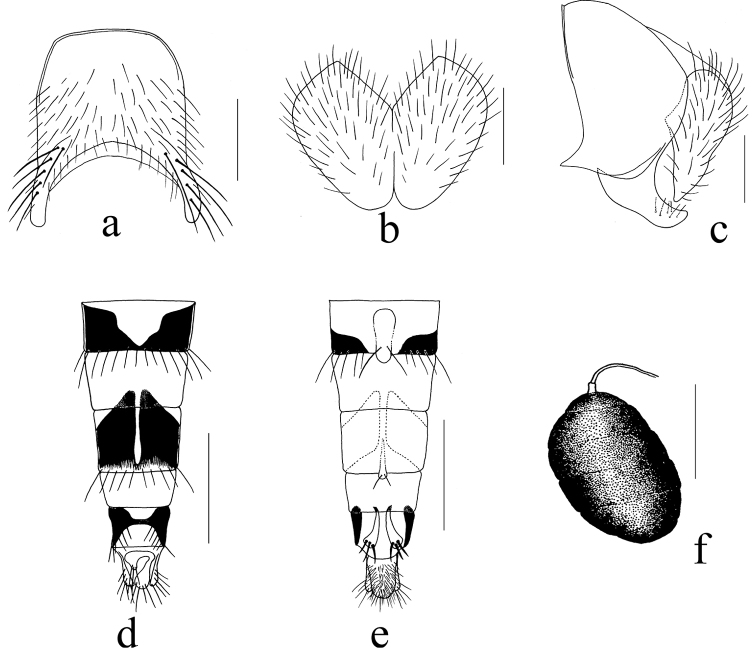
*Mydaea adhesipeda* Xue, sp. nov. **a** male, sternite 5 in ventral view **b** male, cerci in posterior view **c** male, terminalia in profile **d** female, ovipositor in dorsal view **e** female, ovipositor in ventral view **f** female, spermatheca. Scale bars: 0.50 mm (**a**); 0.20 mm (**b,c**); 0.20 mm (**d,e**); 0.10 mm (**f**).

**Female.** Body length 8.9–9.5 mm. Frontal vitta 3.5 times as wide as fronto-orbital plate; 7 frontal setae; 2 upper orbital setae; genal height approximately 1/6 of eye height; ventral surface of fore tarsus without adhesive hairs; mid tibia with 3 or 4 *p*; fore claws and pulvilli shorter than tarsomere 5. Other characters as in male.

#### Remarks.

This species is similar to *Mydaea fuchaoi* Xue & Tian, but differs from the latter in the following features: frontal vitta narrower; genae approximately 1/9 of eye height; palpi shorter; fore claws and pulvilli longer, fore tibia with 1 median *p*; ventral surface of fore tarsus with adhesive hairs; hind claws and pulvilli shorter, hind tibia with 4 *av*; middle part of lateral margin of cerci slightly concave in posterior view.

#### Etymology.

The species name refers to ventral surface of the male fore tarsus which has adhesive hairs. It is derived from the Latin words *adhes* meaning adhesive and *ped* meaning leg.

#### Distribution.

China, Ningxia Hui Autonomous Region (Liupan Mountains).

### Mydaea combiniseriata

Taxon classificationAnimaliaDipteraMuscidae

Xue sp. nov.

A8F2A3E2-D58F-5B7C-A036-BD7476699F1F

http://zoobank.org/CD4832B2-CB16-4CB4-9672-F78F044B8C2F

#### Type material.

***Holotype*.** China, 1 ♂, Liaoning Province, Qingyuan, 41°81'N, 124°91'E, alt. 800 m, 3 June 2016, Bing Li, (SYNU). ***Paratypes*.** 3♂♂, same data as holotype.

#### Diagnosis.

5 or 6 frontal setae situated on lower half of frons; scutellum yellow; postpronotal lobe black; anterior spiracle fuscous; legs with femora and tibiae yellow; only distal part of hind femur with distinct *pv* and 5 or 6 small *pv* on basal half.

#### Description.

**Male.** Body length 7.2–7.4 mm. ***Head***: eyes bare, frontal vitta black, fronto-orbital plates and parafacials brown, mediane red-brown; antennae black, arista brown-yellow; lunule dark brown; genae black, genal and postgenal hairs entirely black; palpi black. Frons less than twice the width of anterior ocellus; fronto-orbital plates contiguous in the middle; frontal triangle on upper 1/3 of frons; fronto-orbital plates and parafacials covered with sparse gray pruinosity, parafacial approximately 1/2 as wide as postpedicel; genal height approximately 1/9 of eye height, genae covered with gray pruinosity; lower face not projecting, vibrissal angle situated behind frontal angle in lateral view. 5or 6 pairs frontal setae situated on lower half of frons; proclinate orbital setae absent; ocellar setae long and strong, slightly longer than the lower frontal setae; postpedicel approximately 3.5 times as long as wide; arista plumose, the longest hairs approximately 4/5 as width of width of postpedicel; palpus black, approximately 1.5 times as long as prementum, prementum short, approximately 1.5 times as long as high, and covered with sparse gray pruinosity; labellum long and big, the length of labellum approximately twice as long as height of prementum. ***Thorax***: fuscous, only scutellum yellow, covered with sparse gray pruinosity; scutum with 4 black vittae, and the inner vittae extending to scutoscutellar suture; *acr* 0+1; *dc* 2+4; *ial* 0+2; *pra* strong, approximately 1.3 times as long as posterior notopleural seta; lateral and ventral surfaces of scutellum without hairs; basisternum of prosternum, anepimeron, meron, katepimeron bare; notopleuron with hairs; katepisternal setae 1+2; anterior spiracle fuscous and posterior spiracle light brown. ***Wings***: semi-hyaline and slightly brown; base of wing pale yellow; tegula and basicosta yellow; costal spine small; ventral surface of vein C with hairs; vein Sc bow-shaped; crossvein r-m straight, crossvein dm-m bent towards base of wing, areas around crossveins r-m and dm-m not clouded; dorsal and ventral surface of radial node with hairs; R_4+5_ and M_1_ straight, apical part of M_1_ bent forward slightly; calypter pale yellow, lower calypter tongue-shaped; halter knob yellow. ***Legs***: femora and tibiae yellow but dorsal surface of fore femur fuscous; coxae, trochanters, and tarsi fuscous; fore tibia without median *p*; mid femur with *pv* row on basal half, 1 apical *ad*, 3 apical *pd*, mid tibia with 3 *p*; hind femur with distinct *av* and *pv* only at distal part, and 5 or 6 small *pv* on basal half; hind tibia with 2 *pd*, 5 *pv*; tarsi slight longer than tibiae; only fore claws longer than pulvilli, mid and hind claws shorter than pulvilli, fore claws approximately as long as tarsomere 5. ***Abdomen***: black in ground color, covered with light gray pruinosity, both sides of abdomen without color shifting patch, tergite 3 with a complete row of posterior marginal setae, but short and sparse, median pair shorter than half the length tergite 3, tergites 4 and 5 with complete rows of posterior marginal setae and these slightly longer, approximately 3/5 of the length of the tergite, tergite 4 with 3 pairs of discal setae, tergite 5 with 4 pairs of discal setae; posterior margin of sternites 2 and 3 each with a pair of apical setae, posterior margin of sternite 4 with 2 pairs of apical setae, inner margin of lateral lobe at basal part of sternite 5 with a row of 6 or 7 close-set setae, sursyli near rectangle, and inner margin with hairs.

**Figure 3.**
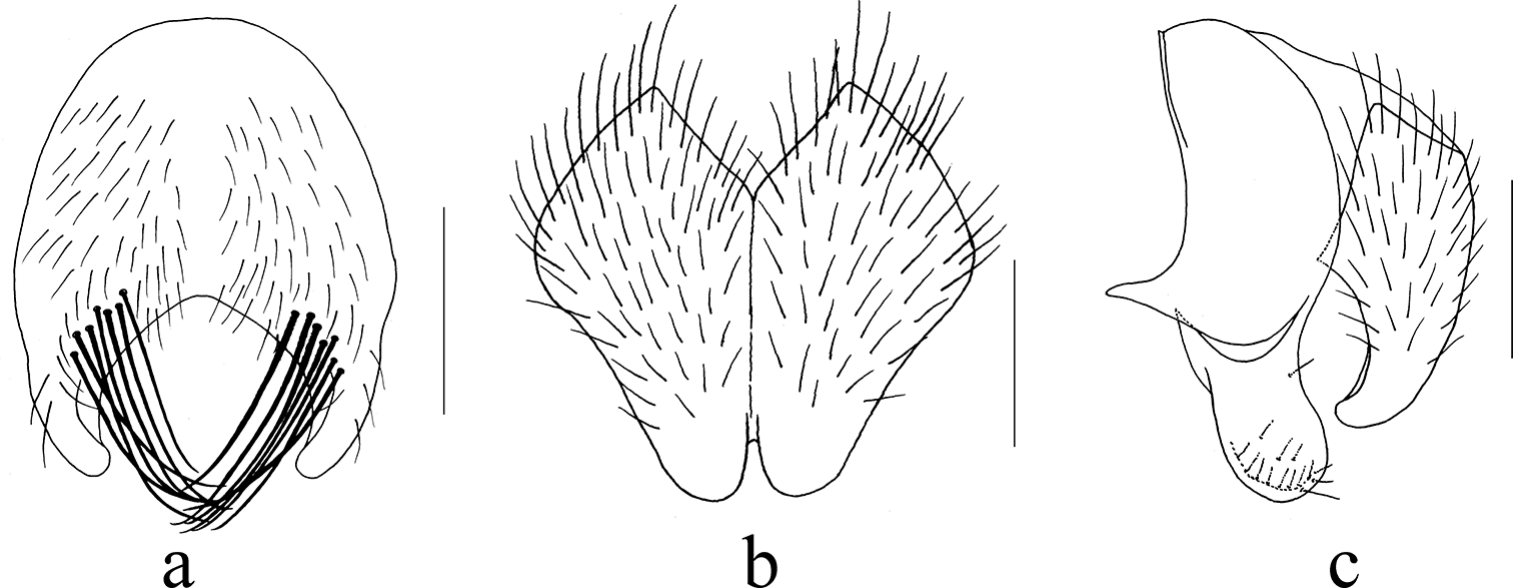
*Mydaea combiniseriata* Xue, sp. nov. **a** male, sternite 5 in ventral view **b** male, cerci in posterior view **c** male, terminalia in profile. Scale bars: 0.50 mm (**a**); 0.25 mm (**b,c**).

**Female.** Unknown.

#### Remarks.

This species is similar to *Mydaea corni* (Scopoli, 1763), but differs from the latter in the following features: male eyes bare; 5 or 6 pairs of frontal setae situated on lower half of frons; genal height approximately 1/9 of eye height; *acr* 0+1; *pra* long and large, approximately 1.3 times as long as posterior notopleural seta; hind tibia without *ad*; both sides of abdomen without color shifting patches.

#### Etymology.

The species name refers to the lobe of sternite 5, which in males have a long row of setae. It is derived from the Latin words *combin* meaning combined and *seriat* meaning rows.

#### Distribution.

China, Liaoning Province (Qingyuan).

### Mydaea qingyuanensis

Taxon classificationAnimaliaDipteraMuscidae

Xue sp. nov.

DB49C7CD-8D1E-5065-BCC7-6800C2FF07AF

http://zoobank.org/A9C2CC5B-E88B-4F91-9B6A-275FE9403DB9

#### Type material.

***Holotype*.** China, 1 ♂, Liaoning Province, Qingyuan, 41°81'N, 124°91'E, alt. 800 m, 3 June 2016, Bing Li, (SYNU). ***Paratypes*.** 2♂♂, same data as holotype.

#### Diagnosis.

Frons narrower than the width of anterior ocellus; scutellum yellow, *prst-acr* absent, *post-dc* 4, anterior and posterior spiracle yellow; hind femur with complete *pv* row, hind tibia without *ad*.

#### Description.

**Male.** Body length 7.8–8.0 mm. ***Head***: eyes bare; frontal vitta black; antennae black,lunule brown; genae black, genal and postgenal hairs entirely black; palpi black. Frons narrower than the width of anterior ocellus; fronto-orbital plates contiguous in the middle; frontal vitta situated on upper 2/5 of frons; Fronto-orbital plates and parafacials covered with sparse gray pruinosity, parafacials approximately 3/5 as wide as postpedicel; lower face not projecting, vibrissal angle situated behind frontal angle in lateral view; genae covered with gray pruinosity, genal height approximately 1/9 of eye height. Frontal setae in 13 or 14 pairs and extending upwards to anterior ocellus, 5 or 6 strong pairs on lower 2/5 of frons, 7 or 8 extremely small pairs on upper 3/5 and these shorter than eye facets ; proclinate orbital setae absent; ocellar setae strong, slightly longer than the lower frontal setae; postpedicel approximately 3.5 times as long as wide, arista plumose, longest hairs approximately as long as width of postpedicel; palpus approximately 1.5 times as long as prementum, prementum short, approximately 1.5 times as long as high, and covered with sparse gray pruinosity; labellum long and big, the length of labellum approximately twice the height of prementum. ***Thorax***: black, but scutellum yellow, covered with sparse gray pruinosity; scutum with 4 black vittae, and inner vitta not extending to scutoscutellar suture; *acr* 0+1; *dc* 2+4; *ial* 0+2; *pra* distinct, slightly shorter than posterior notopleural seta; lateral and ventral surfaces of scutellum without hairs; basisternum of prosternum, anepimeron, meron, and katepimeron bare; notopleuron with hairs; katepisternal setae 1+2; anterior and posterior spiracles yellow. ***Wings***: semi-hyaline and pale brown; tegula and basicosta yellow; costal spine slightly short than crossvein r-m; ventral surface of vein C with hairs; vein Sc bow-shaped; areas around crossveins r-m and dm-m not clouded and straight; dorsal and ventral surface of radial node with hairs; R_4+5_ and M_1_ straight, apical part of M_1_ slightly bent forward; calypter yellow, lower calypter tongue-shaped; halter knob yellow. ***Legs***: Tibiae and femora brown-yellow; coxae, trochanters and tarsi dark brown; fore tibia without median *p*; mid femur without distinct *av*, 1 apical *ad*, 3 apical *pd*, complete *pv* rows, mid tibia with 3 *p*; hind femur with complete *av* and *pv* rows, hind tibia with 2 *pd*, 2 *pv*; tarsi slight longer than tibiae; pulvilli longer than claws, pulvilli approximately 2/3 of tarsomere 5 in length. ***Abdomen***: ground color black; ovate in dorsal view, tergites 3–5 with a median black vitta, both sides of tergites 4 and 5 with distinct color shifting patches, tergites 3–5 with a complete posterior marginal rows of setae, tergites 4 and 5 with 4 or 5 pairs of discal setae, sternite 1 bare, apical margin of sternites 2–4 each with a pair of marginal setae; distal part of cerci and sursyli tapering in posterior view, dispart of cerci triangular in posterior view, sursyli narrowed on distal half in profile and bent backwards.

**Figure 4.**
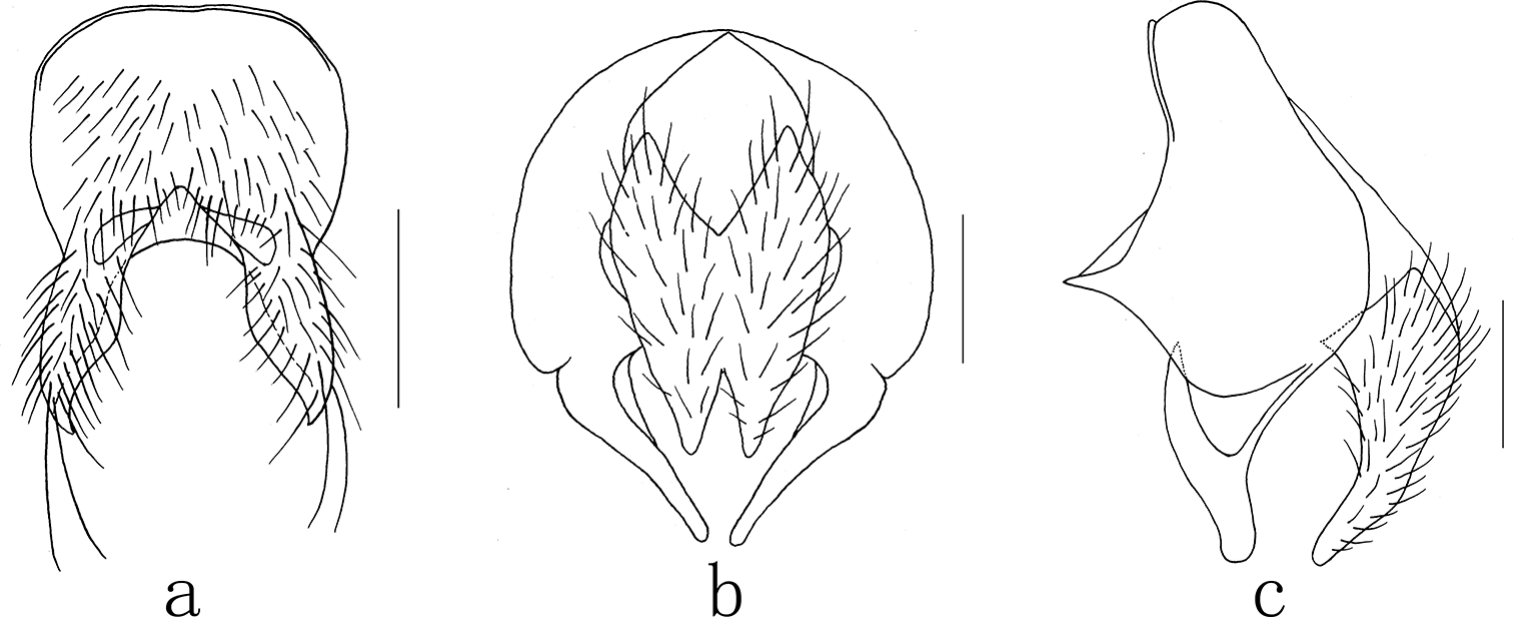
*Mydaea qingyuanensis* Xue, sp. nov. **a** male, sternite 5 in ventral view **b** male, terminalia in posterior view **c** male, terminalia in profile. Scale bars: 0.50 mm (**a**); 0.20 mm (**b**); 0.25 mm (**c**).

**Female.** Unknown.

#### Remarks.

This species is similar to *Mydaea corni* (Scopoli, 1763), but differs from it in the following features: male eyes bare; frontal setae in 13 or 14 pairs and extending upwards to anterior ocellus, 5 or 6 strong pairs on lower 2/5 of frons, 7 or 8 extremely small pairs at upper 3/5 and these shorter than facets of eye; postpedicel approximately 3.5 times as long as wide; genal height approximately 1/9 of eye height; *acr* 0+1; *dc* 2+4; *pra* shorter than posterior notopleural seta; posterior spiracle yellow; hind tibia without *ad*.

#### Etymology.

The species is named for the type locality, Qingyuan county.

#### Distribution.

China, Liaoning Province (Qingyuan).

### Mydaea quinquiseta

Taxon classificationAnimaliaDipteraMuscidae

Xue sp. nov.

E829D4E3-29B6-558B-8FDD-C0FC587C57C8

http://zoobank.org/18E8DE94-D3F6-49AB-A66C-C545926A3CC0

#### Type material.

***Holotype*.** China, 1 ♂, Sichuan Province, Luding, Yanzigou, 29°38'N, 102°07'E, alt. 2600 m, 17 June 2006, Jiayu Liu, (SYNU). ***Paratypes*.** 2♂♂, same data as holotype.

#### Diagnosis.

Eyes bare; *post-dc* 4; distal and lateral part of scutellum yellow; anterior and posterior spiracle brown; legs brown-yellow; both sides of abdomen without color shifting patch; cerci circular apically in posterior view.

#### Description.

**Male.** Body length 7.4–7.6 mm. ***Head***: eyes bare, facets on upper half not enlarged; frontal vitta black; antennae black, arista brown; lunule brown; genae black, genal and postgenal hairs entirely black; palpi black. Frons approximately 1.5 times as wide as anterior ocellus; fronto-orbital plates contiguous in the middle; frontal vitta triangle on upper 1/4 of frons; fronto-orbital plates and parafacial covered with distinct gray pruinosity, parafacial approximately 3/5 as wide as postpedicel; postpedicel approximately 3.5 times as long as wide; lower face not projecting, vibrissal angle situated behind frontal angle in lateral view; genae covered with gray pruinosity, genal height approximately 1/9 of eye height. Frontal setae in 13 or 14 pairs and these situated on lower half of frons; proclinate orbital setae absent; ocellar setae long and strong, slightly longer than the lower frontal setae; arista short plumose, maximum length of hairs approximately 2/3 of the width of postpedicel; palpus approximately 1.5 times as long as prementum, prementum approximately 2.2 times as long as high, and covered with gray pruinosity; labellum strong, extending to posterior part, and slightly longer than palpi. ***Thorax***: black, but distal and lateral parts of scutellum yellow, covered with sparse gray pruinosity; scutum with 4 indistinct vittae and the inner vittae not extending to scutoscutellar suture; *acr* 0+1; *dc* 2+4; *ial* 0+2; *pra* long and strong, slightly longer than posterior notopleural seta; notopleuron with small hairs; lateral and ventral surfaces of scutellum without hairs; basisternum of prosternum, anepimeron, meron and katepimeron bare; katepisternal setae 1+2; anterior and posterior spiracles brown. ***Wings***: semi-hyaline and basal part brown; tegula and basicosta yellow; costal spine small; ventral surface of vein C with hairs; Sc bow-shaped; middle part of crossvein dm-m bent towards base of wing, area around crossveins r-m and dm-m not clouded; dorsal and ventral surfaces of radial node with hairs; R_4+5_ and M_1_ straight, apical part of M_1_ bent forward slightly; calypter slightly brown; lower calypter tongue-shaped; halter knob yellow. ***Legs***: entirely black; fore tibia without median *p*; mid femur with a row of *pv* on basal 3/5, and a row of *a* setae on basal half, 1 apical *ad*, 3 apical *pd*, mid tibia with 3 *p*; hind femur with a distinct row of *av* on distal half, and *pv* on basal half shorter than transverse diameter of hind femur, hind tibia with 4 or 5 *av*, 2 *ad*, without *pv*; claws as long as pulvilli, and short than tarsomere 5. ***Abdomen***: ground color black; ovate in dorsal view, covered with gray pruinosity, both sides without color shifting patch, tergites 3 and 4 with median black vittae, tergites 4 and 5 each with a complete row of posterior marginal setae, tergite 5 with lateral discal setae on anterior half and otherwise devoid of hairs, sternite 1 bare, sternite 5 with 5 strong, median setae; cerci circular apically in posterior view.

**Figure 5.**
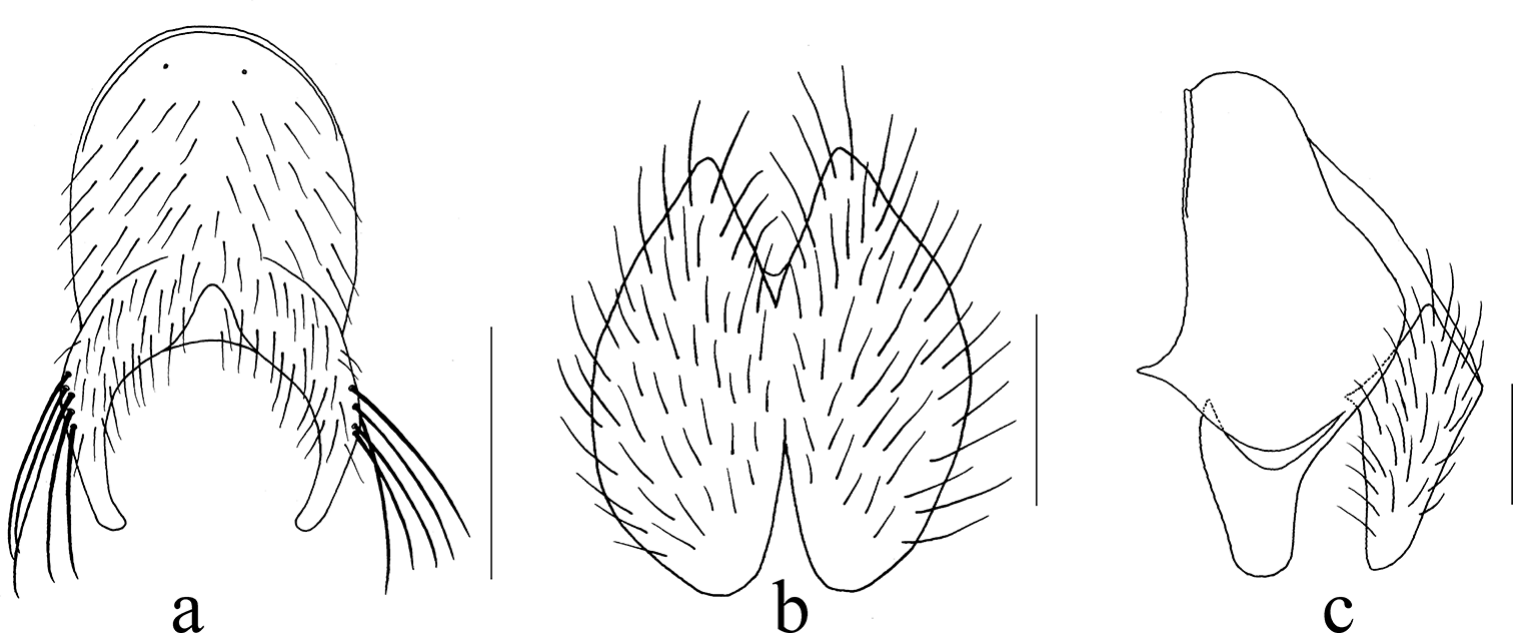
*Mydaea quinquiseta* Xue, sp. nov. **a** male, sternite 5 in ventral view **b** male, cerci in posterior view **c** male, terminalia in profile. Scale bars: 0.50 mm (**a**); 0.20 mm (**b,c**)

**Female.** Unknown.

#### Remarks.

This species is similar to *Mydaea gracilior* Xue, 1992, but differs from the latter in the following features: facets on upper half of eyes not enlarged; *acr* 0+1; *pv* on basal half of hind femur shorter than transverse diameter of hind femur; cersci circular apically in posterior view; distal parts of cerci and surstyli not bent in opposite directions.

#### Etymology.

The species name refers to the lobe of sternite 5 which in males has 5 strong setae. It is derived from the Latin words *quinqu* meaning five and *seta* meaning setae.

#### Distribution.

China, Sichuan Province (Yanzigou).

### Mydaea wusuensis

Taxon classificationAnimaliaDipteraMuscidae

Xue sp. nov.

B6D33F70-5B96-5EA7-A106-946B3E6A7DEF

http://zoobank.org/5A203D16-965B-4634-BE42-03552051AF28

#### Type material.

***Holotype*.** China, 1 ♂, Heilongjiang Province, Jiamusi, Wusuzhen, 48°15'N, 134°12'E, alt. 80 m, 18 May 2017, Bo Hao, (SYNU). ***Paratypes*.** 2♂♂, same data as holotype.

#### Diagnosis.

Scutellum and legs black; hind femur without *pv*; lateral lobes of sternite 5 short, and basal part near quadrate; cerci distinctly narrowed on distal half in posterior view and rounded on apical part, distal parts of cerci and surstyli bent in opposing directions; in lateral view only surstyli bent posteriorly.

#### Description.

**Male.** Body length 5.4–5.6 mm. ***Head***: eyes bare, frontal vitta black; antenna black, arista brown; lunule brown; genae black, genal and postgenal hairs entirely black; palpi black. Frons approximately 1.5 times as wide as anterior ocellus; fronto-orbital plates contiguous in the middle; frontal vitta triangle on upper 1/3 of frons; fronto-orbital plates and parafacial covered with sparse gray pruinosity, parafacials approximately 2/5 width of postpedicel; lower face not projecting, vibrissal angle situated behind frontal angle in lateral view; genae covered with gray pruinosity, genal height approximately 1/9 of eye height; Frontal setae in 7–8 pairs and these situated on lower 3/5 of frons; proclinate orbital setae absent; ocellar setae long and strong, slightly longer than lower frontal setae; postpedicel approximately 3.0 times as long as wide; arista short plumose, maximum length of hairs approximately 2/3 of width of postpedicel; palpi approximately 1.2 times as long as prementum, prementum approximately 2.2 times as long as high, and covered with gray pruinosity; labellum long and large, extending to posterior part and approximately as long as palpi. ***Thorax***: ground color black; covered with sparse gray pruinosity; scutum with 4 black vittae, and the inner vittae not extending to scutoscutellar suture; *acr* 0+1; *dc* 2+4; *ial* 0+2; *pra* approximately as long as posterior notopleural seta; notopleuron with small hairs; scutellum same color as thorax and lateral and ventral surfaces of scutellum without hairs; apical scutellar setae strong, slightly longer than posterior notopleural seta; basisternum of prosternum, anepimeron, meron and katepimeron bare; katepisternal setae 1+2; anterior and posterior spiracles brown. ***Wings***: semi-hyaline, tegula black; basicosta yellowish brown; costal spine short; vein Sc bow-shaped; crossvein dm-m straight, areas around crossveins r-m and dm-m not clouded; dorsal and ventral surfaces of radial node with hairs; R_4+5_ and M_1_ straight; calypter light brown, lower calypter tongue-shaped; halter knob brown-yellow. ***Legs***: entirely black; fore tibia without median *p*; mid femur with *pv* and *a* rows of setae on basal half, 1 apical *ad*, 1 *av* on basal part, 2 apical *pd*, mid tibia with 3 *p*; hind femur with a distinct row of *av* on basal 2/5, without *pv*, hind tibia with 2 *av*, 2 *ad*, without apical *pv*; claws as long as pulvilli, and shorter than tarsomere 5. ***Abdomen***: ground color black; ovate in dorsal view, covered with gray pruinosity, both sides without color shifting patch, tergites 3–5 with a median black vitta, tergites 4 and 5 each with a complete row of posterior marginal setae, tergite 5 with 4 or 5 discal setae; sternite 1 bare, lateral lobe sternite 5 short, and basal part near quadrate; cerci narrowing distinctly in posterior view and rounded on apical part, distal parts of cerci and surstyli bent in opposing directions; in lateral view only surstyli bent posteriorly.

**Figure 6.**
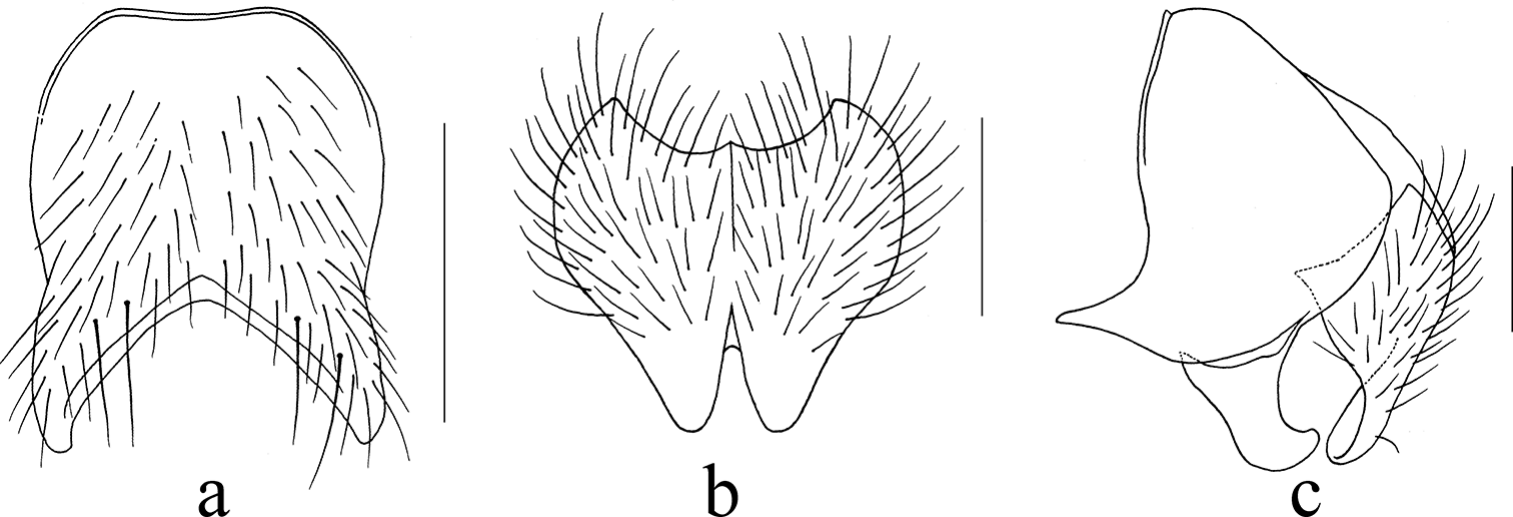
*Mydaea wusuensis* Xue, sp. nov. **a** male, sternite 5 in ventral view **b** male, cerci in posterior view **c** male, terminalia in profile. Scale bars: 0.50 mm (**a**); 0.20 mm (**b,c**).

**Female.** Unknown.

#### Remarks.

This species is similar to *Mydaea ancilloides* Xue, 1992, but differs from it in the following features: male arista short-plumose, longest hair shorter than width of postpedicel; lower 3/5 of frons without frontal setae; parafacial approximately 2/5 of postpedicel in width; sternite 5 lateral lobe short and basal part near quadrate.

#### Etymology.

The specific name refers to its type locality, Wusu town.

#### Distribution.

China, Heilongjiang Province (Wusuzhen).

## Supplementary Material

XML Treatment for Mydaea

XML Treatment for Mydaea adhesipeda

XML Treatment for Mydaea combiniseriata

XML Treatment for Mydaea qingyuanensis

XML Treatment for Mydaea quinquiseta

XML Treatment for Mydaea wusuensis
